# Blockade of IL-6/IL-6R Signaling Attenuates Acute Antibody-Mediated Rejection in a Mouse Cardiac Transplantation Model

**DOI:** 10.3389/fimmu.2021.778359

**Published:** 2021-10-28

**Authors:** Maolin Ma, Qipeng Sun, Xiujie Li, Gengguo Deng, Yannan Zhang, Zhe Yang, Fei Han, Zhengyu Huang, Youqiang Fang, Tao Liao, Qiquan Sun

**Affiliations:** ^1^ Organ Transplantation Research Institute, the Third Affiliated Hospital of Sun Yat-sen University, Guangzhou, Guangdong, China; ^2^ Department of Kidney Transplantation, Guangdong Provincial People's Hospital, Guangdong Academy of Medical Sciences, Guangzhou, Guangdong, China; ^3^ Department of Obstetrics and Gynecology, the Third Affiliated Hospital of Sun Yat-sen University, Guangzhou, Guangdong, China; ^4^ Department of Urology, the Third Affiliated Hospital of Sun Yat-sen University, Guangzhou, Guangdong, China

**Keywords:** antibody-mediated rejection, IL-6, IL-6R, mouse model, cardiac transplantation

## Abstract

Acute antibody-mediated rejection (AAMR) is an important cause of cardiac allograft dysfunction, and more effective strategies need to be explored to improve allograft prognosis. Interleukin (IL)-6/IL-6R signaling plays a key role in the activation of immune cells including B cells, T cells and macrophages, which participate in the progression of AAMR. In this study, we investigated the effect of IL-6/IL-6R signaling blockade on the prevention of AAMR in a mouse model. We established a mouse model of AAMR for cardiac transplantation *via* presensitization of skin grafts and addition of cyclosporin A, and sequentially analyzed its features. Tocilizumab, anti-IL-6R antibody, and recipient IL-6 knockout were used to block IL-6/IL-6R signaling. We demonstrated that blockade of IL-6/IL-6R signaling significantly attenuated allograft injury and improved survival. Further mechanistic research revealed that signaling blockade decreased B cells in circulation, spleens, and allografts, thus inhibiting donor-specific antibody production and complement activation. Moreover, macrophage, T cell, and pro-inflammatory cytokine infiltration in allografts was also reduced. Collectively, we provided a highly practical mouse model of AAMR and demonstrated that blockade of IL-6/IL-6R signaling markedly alleviated AAMR, which is expected to provide a superior option for the treatment of AAMR in clinic.

## Introduction

Cardiac transplantation is the primary treatment for patients with heart failure and has achieved great success (1); however, the median survival of transplant recipients is only 11 years (2). Current immunosuppression strategies have remarkably decreased T cell-mediated rejection (CMR), but antibody-mediated rejection (AMR) is common and has recognized as an important cause of lethal allograft loss (3–5).

AMR is induced by donor-specific antibody (DSA), which can exist preoperatively or *de novo* produced postoperatively. Once DSA binds to the graft vascular endothelial cell surface antigen, complement system is activated to form a membrane attack complex and injure the allografts, to which immune cells, including macrophages and T cells, are recruited, thereby aggravating the injury (6). Therefore, DSA, B cells, macrophages, and T cells plays an important role in the process of AMR. Current therapeutic strategies for AMR mainly include elimination of DSA, depletion of B and plasma cells, and inhibition of complement activation (7). However, these treatments are only partially effective, and may cause severe complications. This situation necessitates the development of a more effective approach for managing AMR.

Therapeutic interventions aimed at blocking cytokine signaling have emerged as an effective strategy for the modification of inflammatory diseases and transplant rejection (8). Interleukin (IL)-6 is an important cytokine that mediates many inflammatory pathways primarily by promoting the expansion and activation of B and T cells (9). Traditional IL-6 signaling is mainly activates two pathways through the IL-6/IL-6R cassette, namely the signaling transducer and activator transcription and mitogen-activated protein kinase pathways, and subsequently activates downstream signals to induce several genes (8, 10). The key role of IL-6 in transplant rejection has been gradually recognized and emphasized. Studies have shown that IL-6 is upregulated in allografts that suffer acute and chronic rejection (11–13). In animal models, blockade of IL-6/IL-6R signaling has been shown to reduce acute CMR and chronic rejection (12, 14). Moreover, the preventive and therapeutic effects and mechanism of the anti-IL-6R antibody tocilizumab on chronic rejection have been explored clinically (15, 16). However, the effects of IL-6/IL-6R signaling on the progression of acute AMR (AAMR) in solid organ transplantation have not been reported.

In this study, we established a mouse cardiac transplantation model for AAMR and sequentially analyzed its features. We then explored the efficacy of blockade of IL-6/IL-6R signaling using tocilizumab and recipient IL-6 knockout (IL-6^-/-^) in suppressing AAMR from allograft survival, pathological changes, DSA, and inflammatory cell infiltration.

## Materials and Methods

### Reagents and Animals

Tocilizumab (Actemra) was purchased from Roche Pharma (Schweiz) Ltd. and dissolved in normal saline. Anti-mouse antibodies including anti-CD3 (ab16669, 1:200), anti-CD4 (D7D2Z, 1:100), anti-CD8 (ab217344, 1:400), anti-mouse C4d (HP8033, 1:200), and anti-CD68 (ab125212, 1:400) were used for immunohistochemical staining. Antibodies used for flow cytometry were AF700-CD45, APC/Cy7-CD3, FITC-CD4, APC-CD8, PC5.5-CD11b, APC-F4/80, and PC5.5-CD19.

Adult male (20–25 g) BALB/c, C57BL/6 wild-type, and IL-6^-/-^ mice were purchased from Charles River Laboratories (Beijing, China) and reared in a specific pathogen-free environment at Sun Yat-sen University. All animal experiments were performed in accordance with the Sun Yat-sen University Institutional Ethical Guidelines and were approved by the Institutional Animal Care and Use Committee.

### Mouse Skin and Cardiac Transplantation

All mice were anesthetized with isoflurane before operation. For skin transplantation (ST), recipient mice were transplanted skin grafts (1 × 1 cm^2^) on their dorsum from donor mice. For cardiac transplantation (CT), donor mice were heparinized, and the heart was exposed by thoracotomy. Then the ascending aorta and pulmonary artery were amputated, the pulmonary veins and the superior and inferior vena cava were ligated. The obtained allograft was stored in cold saline. The recipient mouse underwent abdominal surgery and separation of the abdominal aorta and vena cava. The explanted heart was then transplanted into the recipient mouse *via* end-to-side vascular anastomosis, the pulmonary artery to the vena cava, and the ascending aorta to the abdominal aorta. After the heart restarted, the abdominal cavity was closed. Allograft survival was detected by direct palpation of the fingers, and rejection was considered complete when cardiac arrest occurred. The recipients were sacrificed by excessive anesthesia at the indicated time points.

### Experimental Protocol and Groups

In the first phase, to establish a mouse model of AAMR and analyze its characteristics, recipients were divided into three groups: (A) the non-sensitized (NS) group, donor BALB/c to recipient C57BL/6 mouse CT without ST, normal saline (0.4 mL/d) was injected subcutaneously from day 0 after CT to allograft loss; (B) the presensitized (PS) group, donor BALB/c to recipient C57BL/6 mouse CT 7 days after ST, normal saline (0.4 mL/d) was injected subcutaneously, from day 0 after ST to allograft loss; and (C) the PS + cyclosporin A (CsA) group, donor BALB/c to recipient C57BL/6 mouse CT 7 days after ST, CsA was injected subcutaneously at a dose of 20 mg/(kg·d), from day 0 after ST to allograft loss or obtained for detection.

In the second phase, the PS + CsA group was recognized as the ideal AAMR model. On the basis of different interventions, recipients were divided into three groups: (D) the control group, wherein 12.5 μl saline intraperitoneal injection was performed twice a week on the basis of the PS + CsA group; (E) the tocilizumab group, wherein tocilizumab intraperitoneal injection (0.25 mg, 12.5 μl) was done twice a week, from day 0 after ST to allograft loss or obtained, on the basis of the control group; and (F) the IL-6^-/-^ group, wherein the recipient mouse was replaced by IL-6^-/-^ mice on the basis of the control group.

### Detection of Circulating DSA

The recipient sera were obtained and the DSA (IgG and IgM) levels were assessed at the indicated time points. Briefly, sera combined with donor splenocytes were incubated for 30 min, then washed, and incubated with anti-mouse IgG and IgM (BioLegend, San Diego, CA, USA) at 4°C for 1 h. CytoFLEX flow cytometry (Beckman Coulter, CA, USA) was used to analyze the cells, and the results were presented using mean fluorescence intensity to reveal the levels of DSA in individual serum.

### Histology

At the indicated time points, allografts were obtained, fixed in formalin, and embedded in paraffin. Tissue samples of cardiac grafts were sectioned at 4 μm, deparaffinized, and rehydrated. The samples were then stained with anti-C4d, anti-CD68, anti-CD3, anti-CD4, anti-CD8, and hematoxylin and eosin (H&E).

### Flow Cytometry

Blood, spleen, and cardiac grafts were obtained at 4 d post-transplantation. Spleens and allografts combined with collagenase 1 and DNAse were digested in Roswell Park Memorial Institute 1640 at 37°C for 1 h. The cells were collected cells were stained with fluorochrome-conjugated antibodies for CD45, CD19, CD11b, F4/80, CD3, CD4, and CD8. CytoFLEX flow cytometry (Beckman Coulter) was used to analyze the cells, and FlowJo software (Tree Star Inc., OR, USA) was used to analyze the data.

### Quantitative Real-Time Polymerase Chain Reaction

Quantitative real-time polymerase chain reaction (qRT-PCR) was performed to measure the levels of pro-inflammatory cytokine mRNA in the grafts. Total RNA was extracted from frozen graft tissue using TRIzol reagent (Invitrogen, Carlsbad, CA, USA) and homogenizer, and the cDNA was reverse-transcribed using PrimeScript RT Master Mix (TaKaRa Bio, Japan). qRT-PCR was performed using SYBR Green I master mix (Roche, Switzerland) in a LightCycler480 system (Roche), with β-actin as the internal reference. The Detailed primers used for qRT-PCR are shown in **Table 1**.

**Table 1:** List of primers used for qPCR.

Gene	Forward primer	Reverse primer
β-actin	AGGCCAACCGTGAAAAGATG	TGGCGTGAGGGAGAGCATAG
IL-6	TGCAAGAGACTTCCATCCAGT	TAAGCCTCCGACTTGTGAAGT
IL-2	TGAGCAGGATGGAGAATTACAGG	GTCCAAGTTCATCTTCTAGGCAC
IFN-γ	AGCTCTTCCTCATGGCTGTT	TTTGCCAGTTCCTCCAGATA
IL-4	AACGAGGTCACAGGAGAAGG	TCTGCAGCTCCATGAGAACA
IL-17A	GGCCCTCAGACTACCTCAAC	TCTCGACCCTGAAAGTGAAGG
VCAM-1	TTGGGAGCCTCAACGGTACT	GCAATCGTTTTGTATTCAGGGGA
TNF-α	GCCTCTTCTCATTCCTGCTTG	GGGTCTGGGCCATAGAACTG
iNOS	GTTCTCAGCCCAACAATACAAGA	GTGGACGGGTCGATGTCAC

### Statistics

GraphPad (GraphPad Software, San Diego, CA, USA) was used for the statistical analysis. All data were normally distributed and expressed as the mean ± standard deviation. Student’s *t*-test was used to analyze the difference between two groups. Survival rates were analyzed using Kaplan-Meier curves and log-rank tests. Values of P < 0.05 were considered statistically significant.

## Results

### Sequential Analysis of AAMR in a Mouse Cardiac Transplantation Model

**Figure 1:**
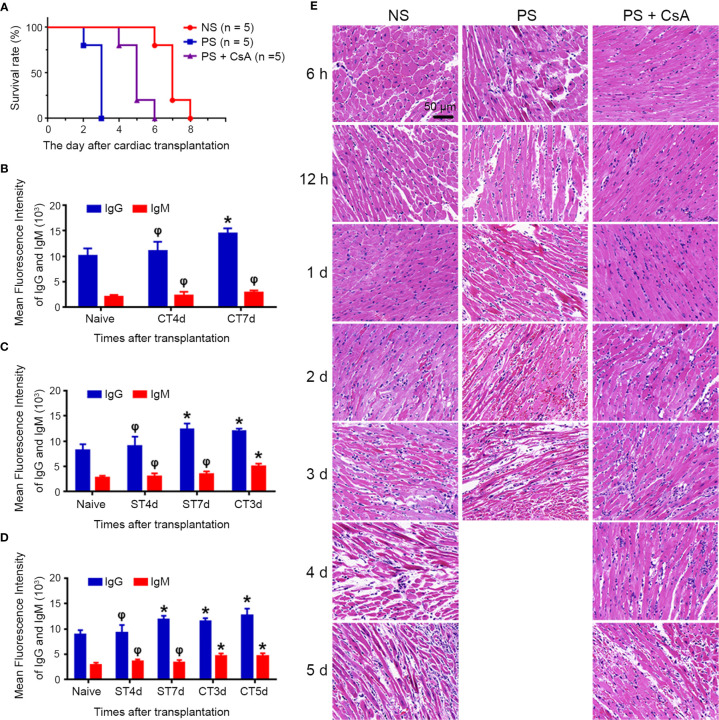
Establishment of mouse model of acute antibody-mediated cardiac allograft rejection. **(A)** Survival rate of the NS, PS and PS + CsA groups (n = 5/group). Sequential analysis of donor-specific antibodies (IgG and IgM) in the NS **(B)**, PS **(C)**, and PS + CsA group **(D)** (n = 5/group). **(E)** Hematoxylin and eosin staining of cardiac allografts in the NS, PS, and PS + CsA groups at indicated time points after cardiac transplantation (n = 5/group). Magnification: 400×; *P<0.05; φ, no significance; NS, non-presensitized; PS, presensitized; PS + CsA, presensitized plus cyclosporine A; ST, skin transplantation; CT, cardiac transplantation.

The NS group in the mouse transplantation model was mainly acute CMR. To establish the AAMR model, we presensitized graft recipients with skin from donors 1 week before cardiac transplantation. Survival time in the PS group was significantly reduced compared to that in the NS group (7.0 ± 0.7 *vs* 2.8 ± 0.4 d, p < 0.01). Considering that skin transplantation activates not only B cells to produce antibodies but also T cells to aggravate CMR, CsA was added to inhibit T cell activation and obtain purer AAMR. Survival time was significantly prolonged after CsA treatment in the PS + CsA group compared to the PS group (5.0 ± 0.7 *vs* 2.8 ± 0.4 d, p < 0.01) (**Figure 1A**).

We performed sequential analysis of DSA (IgG and IgM) during the observation period in each group before cardiac arrest. In the NS group, DSA-IgG was not significantly elevated until 7 d after cardiac transplantation (10040 ± 3343 *vs* 14420 ± 2344, p < 0.05), while DSA-IgM did not increase significantly during the observation period (**Figure 1B**). In the PS and PS + CsA groups, DSA-IgG significantly increased 7 d after skin transplantation (8220 ± 2630 *vs* 12380 ± 2525, p < 0.05; 8880 ± 2018 *vs* 11860 ± 1708, p < 0.05, respectively), and cardiac transplantation was performed at this time point to induce AAMR. Furthermore, DSAs were significantly elevated after cardiac transplantation in the PS and PS + CsA groups (**Figures 1C, D**).

Then, histological features in each group were sequentially observed at the indicated timepoints before cardiac allograft arrest. In the NS group, minimal inflammation in capillaries was first observed in the 2-d allograft and progressed over time. In contrast, in the PS and PS + CsA groups, capillaritis began 6 h after transplantation and was much more severe in the PS group than PS + CsA group at the same timepoints. In addition, in the NS group, interstitial edema and necrosis were first observed at 3 d post-transplantation, and hemorrhage and vascular thrombosis were first observed 4 d post-transplantation. In the PS group, interstitial edema and vascular thrombosis were observed as early as 12 h post-transplantation, and hemorrhage and necrosis were observed 1 d post-transplantation and progressed over time. Meanwhile, the appearance of these pathological features was significantly delayed and noticeably milder in the PS + CsA group than in the PS group (**Figure 1E**).

**Figure 2:**
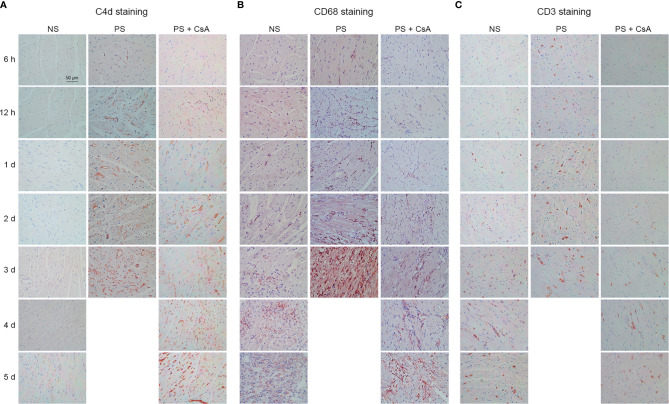
C4d, CD68, and CD3 staining of cardiac allografts. C4d **(A)**, CD68 **(B)**, and CD3 **(C)** staining of cardiac allografts in the NS, PS, and PS + CsA groups at different timepoints after transplantation (n = 5/time point). Magnification: 400×; NS, non-presensitized; PS, presensitized; PS + CsA, presensitized plus cyclosporine A.

To further understand this AAMR mouse model, C4d, CD68, and CD3 in each group were stained. Weakly positive C4d deposition did not appear until 5 d post-operatively in the NS group. In the PS group, focal C4d deposition was seen in 6-h allograft tissue, and all samples after 12 h showed diffuse staining for C4d that increased with time. In the PS + CsA group, C4d staining was weakly positive at 12 h and 1 d post-transplantation, and all allograft samples after 2 d exhibited diffuse, bright staining of C4d that increased with time (**Figure 2A**). Intravascular CD68 staining was initiated 2 d after transplantation in the NS and PS + CsA groups, and this feature was more severe in the PS + CsA group than in the NS group at the same time points. In contrast, intravascular CD68 staining was observed as early as 12 h post-transplantation in the PS group (**Figure 2B**). CD3 staining was first observed at 1 d in the NS group and 2 d in the PS + CsA group after transplantation, and this feature was more severe in the NS group than in the PS + CsA group at the same time points. In contrast, CD3 staining was observed as early as 6 h post-transplantation in the PS group (**Figure 2C**). Moreover, we summarized the histological features in **Table 2** according to the International Society of Heart and Lung Transplantation criteria.

**Table 2:** Histological evaluation according to the criteria of ISHLT.

Histology	Time after cardiac transplantation							
		6h	12h	1d	2d	3d	4d	5d
Capillary C4d distribution (grade)	NS	0	0	0	0	0	0	1 ± 0.7
	PS	0	1.4 ± 0.5	2	2	2		
	PS + CsA	0	0.4 ± 0.5	1	1.4 ± 0.5	1.8 ± 0.4	2	2
Intravascular CD68 distribution (grade)	NS	0	0	0	0.2 ± 0.4	0.2 ± 0.4	0.6 ± 0.5	0.8 ± 0.4
	PS	0	0.6 ± 0.5	0.8 ± 0.4	1.4 ± 0.5	2		
	PS + CsA	0	0	0	0.4 ± 0.5	0.8 ± 0.4	1.4 ± 0.5	2
Intravascular activated mononuclear cells	NS	–	–	–	+	+	+	+
	PS	–	+	+	+	+		
	PS + CsA	–	–	+	+	+	+	+
Capillaritis	NS	–	–	–	+	+	+	+
	PS	+	+	+	+	+		
	PS + CsA	+	+	+	+	+	+	+
Interstitial edema	NS	–	–	–	–	+	+	+
	PS	–	+	+	+	+		
	PS + CsA	–	–	–	+	+	+	+
Hemorrhage	NS	–	–	–	–	–	+	+
	PS	–	–	+	+	+		
	PS + CsA	–	–	–	–	+	+	+
Necrosis	NS	–	–	–	–	+	+	+
	PS	–	–	+	+	+		
	PS + CsA	–	–	–	+	+	+	+
Vascular thrombosis	NS	–	–	–	–	–	+	+
	PS	–	+	+	+	+		
	PS + CsA	–	–	–	–	+	+	+

ISHLT, International Society of Heart and Lung Transplantation; NS, nonpresensitized; PS, presensitized; CsA, Cyclosporine A; −, negative; +, positive.

The scoring criteria of C4d deposition and intravascular CD68 distribution: grade 0, <10% of all capillaries; grade 1, 10%-50% of all capillaries; grade 2, >50% of all capillaries.

Data are mean ± standard deviation.

Histological evaluations were performed using light microscopy by an independent observer, who was blinded to the experimental conditions.

### Blockade of IL-6/IL-6R Signaling Attenuates AAMR and Prolongs Allograft Survival

**Figure 3:**
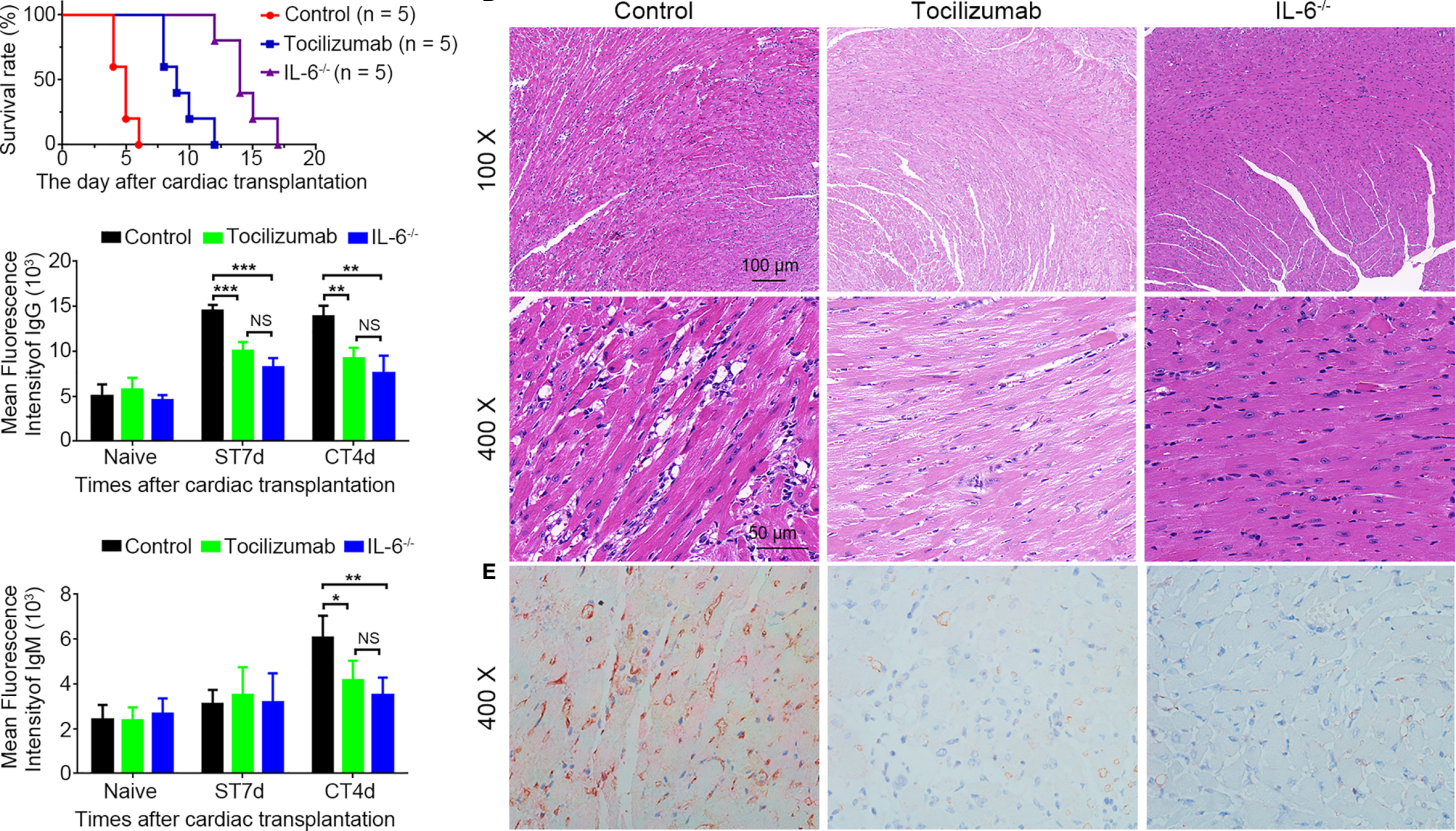
Blockade of IL-6/IL-6R signaling attenuates acute antibody-mediated rejection. **(A)** Survival rate in the control, tocilizumab, and IL-6^-/-^ groups (n = 5/group). **(B, C)** Changes in donor-specific antibody IgG and IgM before and after transplantation in each group (n = 4/group). **(D, E)** Representative hematoxylin and eosin staining and C4d immunohistochemistry staining of cardiac allografts obtained 4 d post-operation in each group (n = 4/group). *P<0.05; **P<0.01; ***P<0.001; ST, skin transplantation; CT, cardiac transplantation. NS, no significance.

The IL-6^-/-^ group had a longer survival time compared to tocilizumab group (14.4 ± 1.8 *vs* 9.4 ± 1.7 days, p < 0.01), and both were significantly longer than that of the control group (14.4 ± 1.8 *vs* 4.8 ± 0.8 d, p < 0.01; 9.4 ± 1.7 *vs* 4.8 ± 0.8 d, p < 0.01; respectively) (**Figure 3A**). For DSA analysis, the tocilizumab and IL-6^-/-^ groups had significantly lower DSA-IgG levels 7 d after skin transplantation and lower DSA (IgG and IgM) 4 d post-operatively compared to the control group (**Figures 3B, C**). Furthermore, H&E and C4d staining showed that tocilizumab treatment and recipient IL-6 knockout significantly attenuated AAMR, including capillaritis, interstitial edema, necrosis, hemorrhage, vascular thrombosis, and C4d deposition (**Figures 3D, E**).

### Blockade of IL-6/IL-6R Signaling Decreases B Cells in Circulation, Spleens, and Allografts

**Figure 4:**
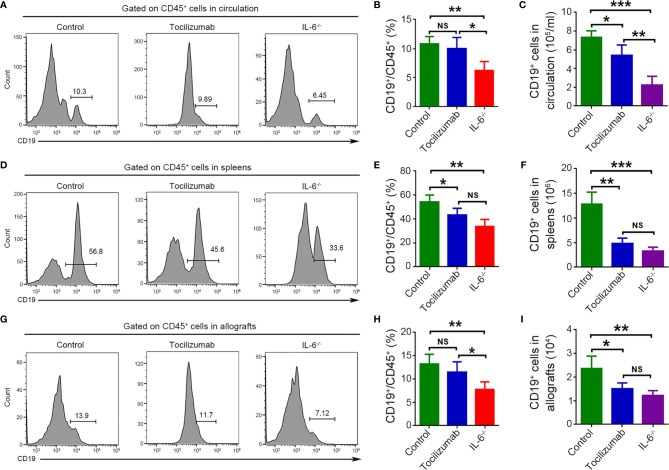
Blockade of IL-6/IL-6R signaling reduces B cells. B cells (CD19^+^) were detected by flow cytometry 4 d after cardiac transplantation in the control, tocilizumab, and IL-6^-/-^ groups. Representative dot plots for frequencies and quantitative analysis of frequencies and counts of B cells in circulation **(A–C)**, spleens **(D–F)**, and cardiac allografts **(G–I)** (n = 4/group). *P<0.05; **P<0.01; ***P<0.001; NS, no significance.

Flow cytometry was used to detect B cells (CD19^+^) in circulation, spleens, and allografts obtained 4 d after transplantation, along with quantitative analysis of the frequencies and cell numbers. In circulation, tocilizumab treatment significantly reduced the absolute number of B cells but did not affect their frequencies. Meanwhile, recipient IL-6 knockout markedly decreased the frequency and number of B cells. Notably, a significantly lower frequency and number of B cells in circulation was observed in the IL-6^-/-^ group compared to that in the tocilizumab group (**Figures 4A–C**). In spleens, tocilizumab treatment and recipient IL-6 knockout markedly decreased B cell number and frequency, and there was no significant difference on frequency and number between the two groups (**Figures 4D–F**). In cardiac allografts, tocilizumab treatment had no significant influence on the frequency of B cells but significantly reduced the B cell number, while recipient IL-6 knockout markedly decreased both the frequency and number of B cells. Moreover, there was a significant difference in frequency but no significant difference in the number of B cells in allografts between the tocilizumab and IL-6^-/-^ groups (**Figures 4G–I**).

### Blockade of IL-6/IL-6R Signaling Reduces Macrophage and T Cell Infiltration in Allografts

**Figure 5:**
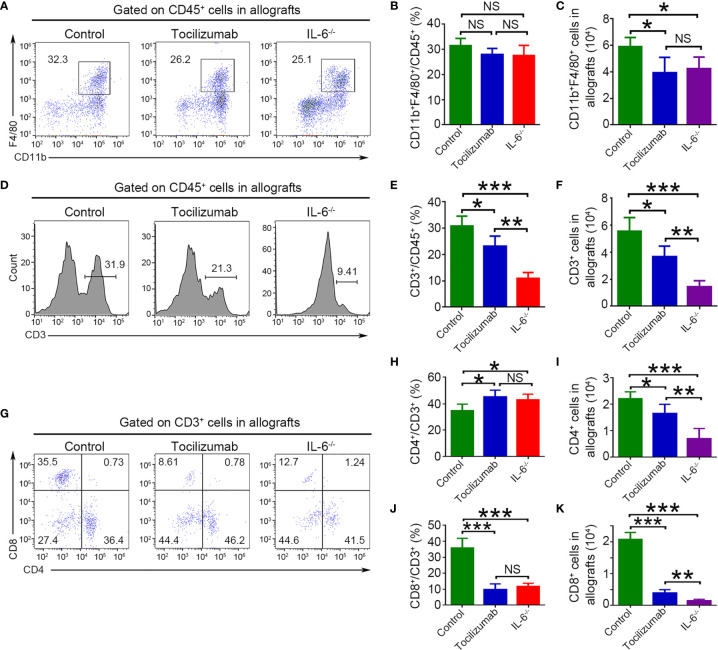
Blockade of IL-6/IL-6R signaling reduces macrophages and T cells infiltration in allografts detected by flow cytometry. Macrophages (CD11b^+^F4/80^+^), T cells (CD3^+^), CD4^+^ T cells (CD3^+^CD4^+^), and CD8^+^ T cells (CD3^+^CD8^+^) were detected in allografts 4 d after transplantation in the control, tocilizumab, and IL-6^-/-^ groups. Representative dot plots for frequencies and quantitative analysis of frequencies and counts of macrophages **(A–C)**, T cells **(D–F)**, CD4^+^ T cells and CD8^+^ T cells **(G–K)** (n = 4/group). *P<0.05; **P<0.01; ***P<0.001; NS, no significance.

Macrophages (CD11b^+^F4/80^+^) and T cells (CD3^+^, CD4^+^, and CD8^+^) in allografts were visualized using flow cytometry. The results showed that tocilizumab treatment and recipient IL-6 knockout markedly decreased the number of macrophages, but had no effect on cell frequencies, and these values were not significantly different between the two groups (**Figures 5A–C**). In CD3^+^ T cells, tocilizumab treatment and recipient IL-6 knockout markedly reduced cell number and frequency, which were significantly different between the two groups (**Figures 5D–F**). Further analysis of T cells revealed that tocilizumab treatment and recipient IL-6 knockout lowered the percentage of CD8^+^ T cells but increased that of CD4^+^ T cells among CD3^+^ cells while reducing the absolute numbers of both CD4^+^ and CD8^+^ T cells. This may be explained by the reduction in CD8^+^ T cells being greater than that in CD4^+^ T cells. In addition, there was a significant difference in number but not in the frequency of CD4^+^ and CD8^+^ T cells between the tocilizumab and IL-6^-/-^ groups (**Figures 5G–K**).

**Figure 6:**
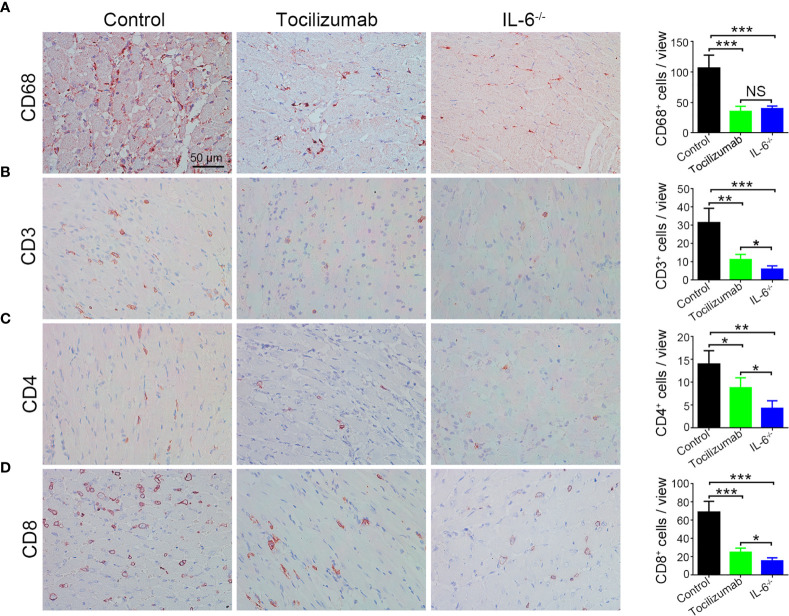
Blockade of IL-6/IL-6R signaling reduces macrophages and T cells infiltration in allografts detected by immunohistochemistry staining. Representative images and quantitative analysis of CD68 **(A)**, CD3 **(B)**, CD4 **(C)**, and CD8 **(D)** in the control, tocilizumab, and IL-6^-/-^ groups (n = 4/group). *P<0.05; **P<0.01; ***P<0.001; NS, no significance.

Moreover, immunohistochemistry results stained CD68, CD3, CD4, and CD8 were consistent with those of flow cytometry (**Figures 6A–D**).

### Blockade of IL-6/IL-6R Signaling Decreases Pro-Inflammatory Cytokines in Allografts

**Figure 7:**
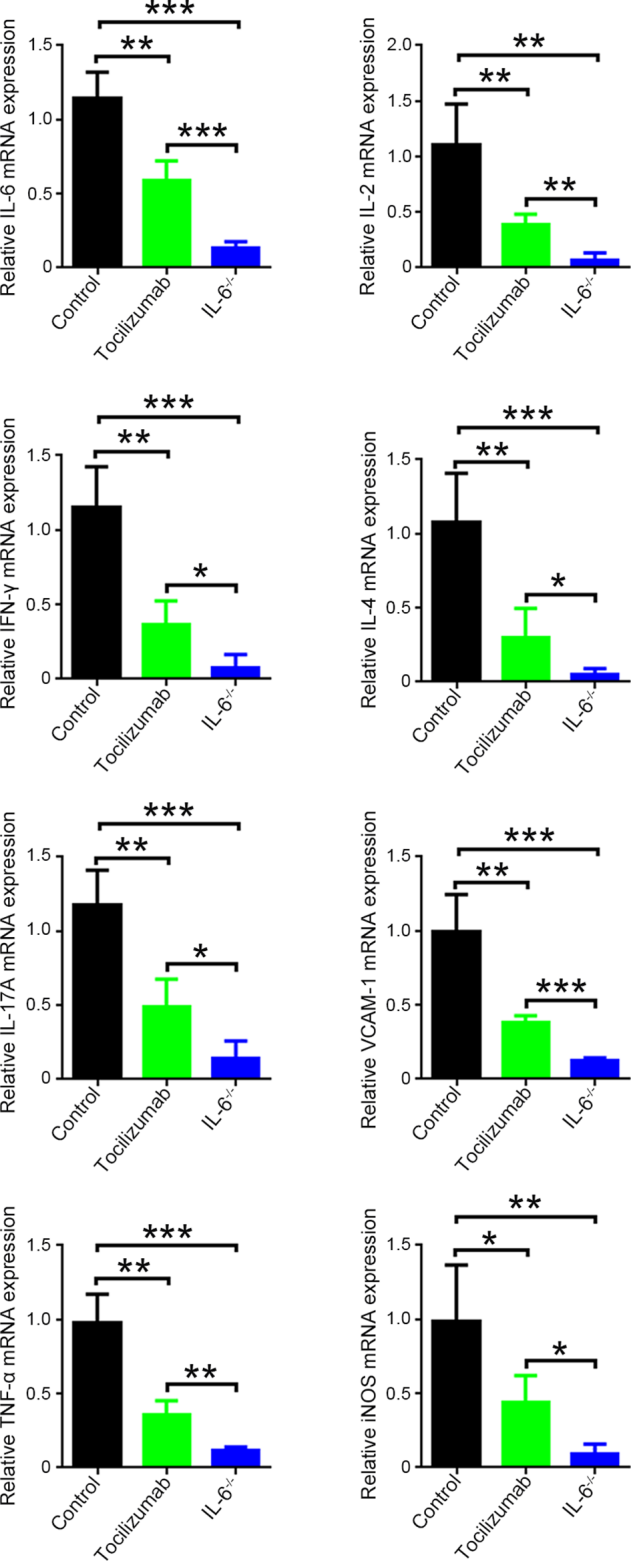
mRNA expression levels of pro-inflammatory cytokines in cardiac allografts. The mRNA expression of *IL-6*, *IL-2*, *IFN-γ*, *IL-4*, *IL-17A*, *VCAM-1*, *TNF-α*, and *iNOS* in cardiac allografts 4 d post-transplantation in the control, tocilizumab, and IL-6^-/-^ groups (n = 4/group). *P<0.05; **P<0.01; ***P<0.001.

We detected *IL-6*, *IL-2*, interferon gamma (*IFN-γ*), *IL-4*, *IL-17A*, vascular cell adhesion molecule 1 (*VCAM-1*), tumor necrosis factor alpha (*TNF-α*), and inducible nitric oxide synthase (*iNOS*) in allografts using qPCR. The results showed a considerable decrease in the expression of these pro-inflammatory cytokines with tocilizumab treatment and recipient IL-6 knockout and this was more evident in the IL-6^-/-^ group than in the tocilizumab group (**Figure 7**).

## Discussion

In this study, we sequentially analyzed AAMR in a mouse cardiac transplantation model and confirmed the remarkable therapeutic effect of blockade of IL-6/IL-6R signaling for AAMR. This effectively attenuated cardiac allograft injury and prolonged survival by inhibiting B cell activation and DSA production, decreasing inflammatory cell infiltration, and reducing inflammatory cytokine release in allografts.

This study provided a mouse cardiac allograft AAMR model and sequentially analyzed its features. Disease studies depend on animal models, and in consideration of this, we have extensive experience in the establishment of mouse and rat AAMR models for renal transplantation and rat AAMR models for cardiac transplantation (17–20). In this study, we initially attempted to establish a mouse AAMR model by priming C57BL/6 recipients with BALB/c donor-specific skin grafts 7 d prior to cardiac transplantation. Graft survival, DSA (IgG and IgM) level, and histology were examined at the indicated timepoints. Allografts were rejected rapidly (2.8 ± 0.4 d), DSA-IgG was elevated during and after cardiac transplantation, and pathological features, such as tissue necrosis, capillaritis, thrombosis, high C4d deposition, and macrophages infiltration in capillaries, rapidly occurred. However, the rejection in this model was too severe and tended to be hyperacute rejection. Considering that presensitization by skin transplantation activates not only B cells to produce DSA but also T cells, we added CsA to alleviate CMR components and obtain a purer and milder AAMR model. In the PS + CsA group, the allograft survival time was 5.0 ± 0.7 d, and DSA-IgG was elevated at the cardiac transplantation timepoint. Moreover, IgG and IgM levels were elevated 3 and 5 d after cardiac transplantation. Histology also showed capillaritis, thrombosis, C4d deposition, and macrophage infiltration in capillaries, but these features were considerably milder than those in the PS group. Furthermore, CD3 infiltration significantly decreased after CsA treatment. Therefore, we believe that the PS + CsA group is an ideal mouse AAMR model that may be used for further research.

The importance of IL-6/IL-6R signaling in transplant rejection has been recognized and highlighted in literature (21). An early study revealed that IL-6 levels were increased in cardiac allografts suffering from chronic rejection with fibrosis, and these effects were significantly reversed by neutralization of IL-6 with monoclonal antibody (12). This study provides the first evidence that blockade of IL-6/IL-6R signaling is a promising treatment strategy for chronic rejection. Subsequently, Stanley C et al. explored the therapeutic effect of tocilizumab against chronic rejection in clinical renal transplantation and achieved good preliminary results (15, 16). Several studies have also demonstrated that blockade of IL-6/IL-6R signaling could inhibit the B cell activation into plasma cells and antibody production (22, 23). These suggest IL-6/IL-6R signaling as a promising target for cytokine intervention in the treatment of AAMR in solid organ transplantation.

We employed two approaches to block IL-6/IL-6R signaling, namely tocilizumab treatment and recipient IL-6 knockout. The results showed that blockade of this signaling pathway significantly attenuated cardiac allograft injury and prolonged survival. Further mechanistic research revealed decrease of B cells in circulation, spleens, and allografts, thus inhibiting DSA production and complement activation. Moreover, macrophage, T cell, and pro-inflammatory cytokine infiltration in allografts were reduced. It is worth noting that the therapeutic effect for AAMR in the IL-6^-/-^ group was significantly better compared to that in the tocilizumab group. This may be explained by the IL-6 knockout resulting in more complete blockage of IL-6/IL-6R signaling than tocilizumab, or by IL-6 acting through mechanisms other than IL-6R.

Recently, the importance of AMR in cardiac transplantation has gained increasing attention for its association with lethal allograft dysfunction and severe coronary arteriosclerosis (24–26). Current therapeutic strategies for AMR mainly aim to eliminate circulating DSA, suppress B and plasma cells, and inhibit complement activation (7). However, these approaches appear to elicit insufficient or transient effects and are associated with severe complications, such as severe infection and toxic effects. Taken together, our results suggest that tocilizumab, a commonly used clinical drug, may be used for the prevention of AAMR and is expected to improve the therapeutic effect against AAMR and the prognosis of allografts.

This study, however, is subject to limitations. First, for AAMR treatment, blockade of IL-6/IL-6R signaling alone was not sufficient for allograft long-term survival, thereby requiring a combination of multiple approaches. Second, the therapeutic efficacy of tocilizumab for AAMR in humans requires further investigation since AAMR in mice are more readily reduced than in humans. Lastly, although we have demonstrated that blockade of IL-6/IL-6R signaling reduces AAMR by inhibiting immune cell activation, the underlying mechanism of its effects on various immune cells, including B cells, T cells, and macrophages, has not been elucidated and warrants further exploration.

Overall, this study established a mouse model of AAMR for cardiac transplantation by presensitization of skin grafts and addition of CsA, and sequentially analyzed its features. We demonstrated that blockade of IL-6/IL-6R signaling markedly alleviated AAMR and may therefore be a superior treatment strategy for AAMR in clinic.

## Data Availability Statement

The raw data supporting the conclusions of this article will be made available by the authors upon reasonable request, without undue reservation.

## Ethics Statement

The animal study was reviewed and approved by Sun Yat-Sen University Institutional Ethical Guidelines.

## Author Contributions

QqS, TL, and YF conceptualized the study. MM, QpS, XL, and TL performed the experiments. GD performed the transplantation model. MM, YZ, and ZY drafted the manuscript. FH and ZH edited and finalized the paper. All authors contributed to the article and approved the submitted version.

## Funding

This study was supported by the Science and Technology Project of Guangzhou City (No. 202102010341), National Natural Science Foundation of China (Nos. 81800663, 82170767, 81800661, 81970650, 81770753 and 81970649), the Guangdong Basic and Applied Basic Research Foundation (Nos. 2019A1515011942, 2021A1515012270 and 2021A1515011379), and the National Key R&D Program of China (No. 2018YFA0108804).

## Conflict of Interest

The authors declare that the research was conducted in the absence of any commercial or financial relationships that could be construed as a potential conflict of interest.

## Publisher’s Note

All claims expressed in this article are solely those of the authors and do not necessarily represent those of their affiliated organizations, or those of the publisher, the editors and the reviewers. Any product that may be evaluated in this article, or claim that may be made by its manufacturer, is not guaranteed or endorsed by the publisher.

## Abbreviations

CMR, T cell-mediated rejection; AMR, antibody-mediated rejection; DSA, donor-specific antibody; IL, interleukin; AAMR, acute antibody-mediated rejection; ST, skin transplantation; CT, cardiac transplantation; NS, non-sensitized; PS, presensitized; CsA, cyclosporin A; H&E, hematoxylin and eosin; IL-6^-/-^, IL-6 knockout; IFN-γ, interferon gamma; iNOS, inducible nitric oxide synthase; qRT-PCR, quantitative real-time polymerase chain reaction; TNF-α, tumor necrosis factor alpha; VCAM-1, vascular cell adhesion molecule 1.
