# Corrigendum: Blockade of IL-6/IL-6R Signaling Attenuates Acute Antibody-Mediated Rejection in a Mouse Cardiac Transplantation Model

**DOI:** 10.3389/fimmu.2021.808329

**Published:** 2021-11-18

**Authors:** Maolin Ma, Qipeng Sun, Xiujie Li, Gengguo Deng, Yannan Zhang, Zhe Yang, Fei Han, Zhengyu Huang, Youqiang Fang, Tao Liao, Qiquan Sun

**Affiliations:** ^1^ Organ Transplantation Research Institute, the Third Affiliated Hospital of Sun Yat-sen University, Guangzhou, Guangdong, China; ^2^ Department of Kidney Transplantation, Guangdong Provincial People’s Hospital, Guangdong Academy of Medical Sciences, Guangzhou, Guangdong, China; ^3^ Department of Obstetrics and Gynecology, the Third Affiliated Hospital of Sun Yat-sen University, Guangzhou, Guangdong, China; ^4^ Department of Urology, the Third Affiliated Hospital of Sun Yat-sen University, Guangzhou, Guangdong, China

**Keywords:** antibody-mediated rejection, IL-6, IL-6R, mouse model, cardiac transplantation

In the original article, there was a mistake in **Figure 2** as published. **In** **Figure 2A** **, C4d staining results in the NS group 1d and 2d have overlapping regions indicating that they are from the same sample. After carefully checking the original data, the image 1d was misused by accident when we were preparing for manuscript submission.** The corrected **Figure 2** appears below.

**Figure 2:**
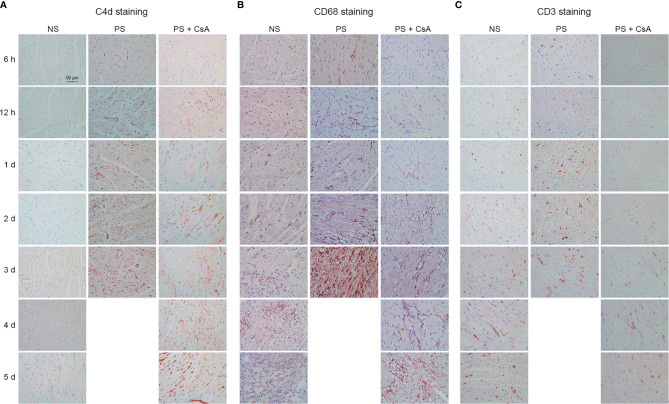
C4d, CD68, and CD3 staining of cardiac allografts. C4d **(A)**, CD68 **(B)**, and CD3 **(C)** staining of cardiac allografts in the NS, PS, and PS + CsA groups at different timepoints after transplantation (n = 5/time point). Magnification: 400×; NS, non-presensitized; PS, presensitized; PS + CsA, presensitized plus cyclosporine A.

The authors apologize for this error and state that this does not change the scientific conclusions of the article in any way. The original article has been updated.

## Publisher’s Note

All claims expressed in this article are solely those of the authors and do not necessarily represent those of their affiliated organizations, or those of the publisher, the editors and the reviewers. Any product that may be evaluated in this article, or claim that may be made by its manufacturer, is not guaranteed or endorsed by the publisher.

